# Future Pose Prediction from 3D Human Skeleton Sequence with Surrounding Situation

**DOI:** 10.3390/s23020876

**Published:** 2023-01-12

**Authors:** Tomohiro Fujita, Yasutomo Kawanishi

**Affiliations:** Guardian Robot Project R-IH, RIKEN, Advanced Telecommunications Research Institute International, 3rd Floor, 2-2-2 Hikaridai, Seika-cho, Sorakugun, Kyoto 619-0288, Japan

**Keywords:** pose prediction, 3D skeleton sequence, surrounding information

## Abstract

Human pose prediction is vital for robot applications such as human–robot interaction and autonomous control of robots. Recent prediction methods often use deep learning and are based on a 3D human skeleton sequence to predict future poses. Even if the starting motions of 3D human skeleton sequences are very similar, their future poses will have variety. It makes it difficult to predict future poses only from a given human skeleton sequence. Meanwhile, when carefully observing human motions, we can find that human motions are often affected by objects or other people around the target person. We consider that the presence of surrounding objects is an important clue for the prediction. This paper proposes a method for predicting the future skeleton sequence by incorporating the surrounding situation into the prediction model. The proposed method uses a feature of an image around the target person as the surrounding information. We confirmed the performance improvement of the proposed method through evaluations on publicly available datasets. As a result, the prediction accuracy was improved for object-related and human-related motions.

## 1. Introduction

Prediction of future human poses and locations from past information is crucial for many tasks, such as autonomous running [1] and risk prediction [2] for a robot. It is also very significant for human tracking [3] and human–robot interaction [4,5]. Even for predicting a short period such as one second or less, it is useful for the assistive robot. In this paper, our goal is to accurately predict the 1-second future poses of a target person from a short-term observation.

Since a time series of sets of body-joint locations in 3D coordinates, namely *3D human skeleton sequences*, are robust to environmental factors such as clothing and background, they are often used for modeling human poses. Machine learning approaches, such as the hidden Markov model [6] and restricted Boltzmann machine [7], are effective for simple motion prediction. However, pose prediction including complex motions is potentially difficult; in recent years, the prediction methods based on deep learning models, such as recurrent neural networks (RNNs) [8,9,10,11,12] and graph convolutional networks (GCNs) [12,13,14,15,16], have been used for accurate prediction.

**Figure 1:**
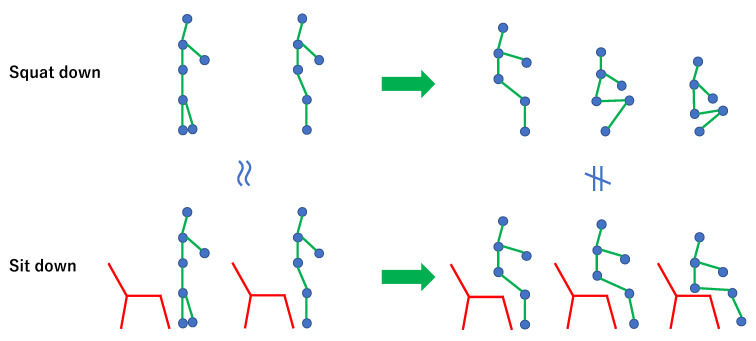
Example of motions that show that starting motions are very similar, but the future motions are different. In such cases, the predictions may be difficult, or the deep learning model may not train well. It is not enough to use only the 3D human skeleton sequence of starting motion.

Most existing studies on human pose prediction use only a short-term 3D human skeleton sequence or a set of 3D human skeleton sequences as an input [8,9,10,11,12,13,14,15,16]. However, it is difficult to predict future poses only from a short-term 3D human skeleton sequence, especially for similar starting motions but different future poses. Figure 1 shows an example of the cases in which the starting motions are very similar while the future poses are different; squatting down and sitting down motions. Additionally, jumping up and picking up are also very similar to the squatting down and sitting down motions. In such cases, problems may arise in that deep learning models cannot be trained well or the results of the prediction will be wrong.

Here, when carefully observing human motions, we can find that human motions are often affected by objects or humans in the surroundings, such as carrying an object and following another person. Based on this fact, we consider that it is important not only to use the 3D human skeleton sequences but also to capture the surrounding information of the target person. In this paper, we propose a prediction method for the future human skeleton sequence from a 3D human skeleton sequence with surrounding information. The method uses an image of human surroundings corresponding to the last frame of the input 3D human skeleton as additional information. Additionally, to effectively utilize the surrounding information, we propose a novel skeleton feature weighting method named the image-assisted attention (IAA) module for future skeleton prediction. It can be inserted into any existing GCN-based prediction method.

Our contributions are summarized as follows:

- We propose a future pose prediction method that captures the surrounding situation of the target person by utilizing an image around the target person at the last frame of the input sequence as additional information.
- We also propose a novel skeleton feature weighting method named image-assisted attention for future skeleton prediction (IAA). It can be applied to any existing GCN-based prediction method to effectively utilize the surrounding information.

The rest of this paper is organized as follows. In Section 2, related work on human pose prediction is summarized. In Section 3, the details of the proposed human pose prediction method and the new feature weighting method are described. In Section 4, experimental results and discussion are presented. Finally, we conclude the paper in Section 5. This paper is a further improvement of our presentation at T-CAP 2022 (ICPR Workshop) [17].

## 2. Related Work

Early studies on the prediction of a 3D human skeleton sequence used machine learning such as the hidden Markov model [6], restricted Boltzmann machine [7], and Gaussian process latent variable models [18]. These traditional machine-learning methods still have difficulty, especially in capturing complex human motions; thus, in recent years, deep-learning-based methods, such as recurrent neural networks (RNNs) and graph convolutional networks (GCNs), have been widely used for future skeleton prediction.

RNNs have several recursive structures inside that allow for processing variable-length input, and they enable the prediction of 3D human skeleton sequences as time-series data [8,9,10,11,12]. Meanwhile, since RNNs are generally difficult to train, and a human body skeleton can be considered as a graph structure, recent studies use GCNs, which can convolve any graph structure, to predict 3D skeleton sequences [12,13,14,15,16]. GCNs are also useful in a wide range of fields such as action recognition [19,20], pose estimation [21,22], and object detection [23,24] using a graphical representation of spatial and temporal information. Furthermore, recently, there has been a prediction method with a manifold-aware generative adversarial network (GAN) to avoid predicting implausible poses and predict smooth motions and plausible poses [25]. Additionally, there is a prediction method using a new GCN architecture called depth-wise graph convolution for reduced parameters and faster prediction [26].

However, the studies of human pose prediction mentioned above use only the short-term human skeleton sequence of the starting motion. These studies do not take into account the situation surrounding the human; thus, if the starting motions of the target person are very similar among different motions, the prediction will be difficult.

For this problem, several studies have proposed skeleton prediction methods that consider the surrounding information. Corona et al. have proposed a prediction method that considers multiple human skeletons and the locations and classes of the other objects [27]. Adeli et al. have proposed a prediction method that utilizes multiple human skeletons and frame images to consider the scene contexts [28]. However, the study by Corona et al. [27] requires somehow selecting an object related to human motions. Additionally, Adeli et al. [28] use only 13 body joints, resulting in a coarse prediction compared to other methods that predict more than 20 joints, calculation costs are high, and the prediction speed is also limited.

Besides the above, Chao et al. [29] used only a static image to predict a 3D skeleton sequence considering future skeleton prediction as a generalization of pose estimation. While the method of Chao et al. [29] takes a single image and requires separate future poses for every time step, Zhang et al. [30] used multiple images from a video for the prediction to recurrently predict a longer range future by taking advantage of a sequence of past images. Compared with these studies, in our study, we propose a prediction method for a 3D human skeleton sequence that uses both an image and a 3D human skeleton sequence and dynamically captures the surrounding situation by using an image feature extracted from a single image around a target person as surrounding information for weighting the skeleton feature. Our method differs in that it uses an image as a supplement, taking advantage of the fact that skeleton-based prediction models are robust to environmental factors and have high prediction accuracy.

## 3. Future Skeleton Prediction That Captures Surrounding Situation

### 3.1. Overview

Existing studies on human skeleton prediction that use only a short-term 3D human skeleton sequence to predict future skeletons cannot accurately predict situations where the starting motions are similar, but the future ones are different. To enable differentiated predictions in such cases, we propose a future skeleton prediction method that captures the surrounding situation by utilizing an image around a target person, named surroundings-aware future skeleton prediction. The proposed method can extend any existing GCN-based future skeleton prediction method that uses a short-term 3D human skeleton sequence by introducing information from the surrounding situation into the GCN.

Here, we assume that the input is a 3D human skeleton sequence $X^{\mathrm{in}}=\left(X_{T_{1}},\ldots ,X_{T_{\mathrm{in}}}\right)$ and the future skeleton sequence $X^{\mathrm{out}}=\left(X_{T_{\mathrm{in}}+1},\ldots ,X_{T_{\mathrm{out}}}\right)$ is to be predicted. For capturing the surrounding situation, the proposed method additionally uses $I_{T_{\mathrm{in}}}$, named the human surrounding image, which is a cropped image around the target person at the time $T_{\mathrm{in}}$ so that more recent surrounding information can be used. Since the view is limited to around the person, the image is expected to contain objects or humans that affect the motion of the target person. To effectively utilize the human surrounding image, we also propose a new feature weighting method named image-assisted attention for future skeleton prediction (IAA), which is a module to modify the skeleton feature extracted from a 3D human skeleton sequence depending on the feature extracted from the human surrounding image.

**Figure 2:**
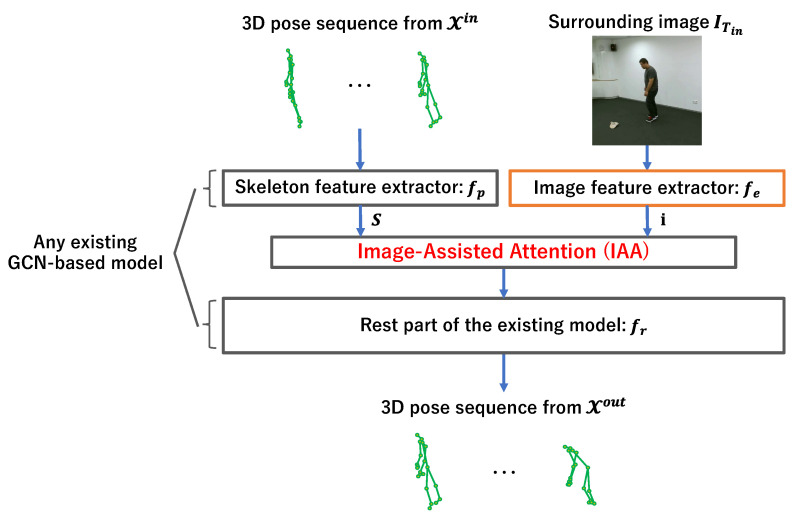
The proposed surrounding-aware future skeleton prediction framework. The skeleton feature *S* is modified using the surrounding image feature i extracted from the human surrounding image.

The framework of the proposed method from input to output is visualized in Figure 2. First, a skeleton feature *S* is extracted from an input 3D human skeleton sequence $X^{\mathrm{in}}$ via the first layers of a GCN-based model. In parallel, a surrounding image feature i is extracted from the human surrounding image $I_{T_{1}}$. Then, the IAA module modifies the skeleton feature *S* by weighting it using the surrounding image feature i. Finally, the modified skeleton feature is fed into the rest part of the GCN-based prediction model to predict the future skeletons $X^{\mathrm{out}}$. The following sections explain the proposed method in detail.

### 3.2. Surrounding Image Feature Extraction

This section explains how to extract the surrounding image feature from the input image. First, given an original RGB image observing the target person, the human surrounding image $I_{T_{\mathrm{in}}}$ corresponding to the human skeleton sequence $X^{\mathrm{in}}$ is cropped. Here, the size of the original image is 1920×1080, and the size of a target person is assumed to be less than about 400×900 pixels in the image. The human surrounding image is cropped via the following steps:

1. The center of the target person in the image coordinate is calculated from the corresponding human skeleton.
2. A 900×900 image centered on the center of the target person is cropped from the 1920×1080 original RGB image.
3. If a part of the cropped image is outside of the original image, the part is filled with 0.

Then, the surrounding image feature is extracted from the cropped image. Here, the extracted feature should capture the existence of objects and humans in the image. Therefore, in this study, we used a pre-trained image feature extractor; specifically, the convolutional layers of EfficientNet [31] pre-trained with the ImageNet. We select the EfficientNet-B3 model empirically, considering the trade-off between accuracy and calculation costs.

Since the size of a human surrounding image is 900×900, it is scaled down to fit the EfficientNet-B3 input size of 300×300 using bilinear interpolation. Then, the surrounding image feature is extracted from the resized human surrounding image by using the pre-trained image feature extractor. Note that the parameters of the feature extractor are not updated during the training of the proposed model in terms of calculation costs to avoid loss of pre-trained parameters.

### 3.3. Image-Assisted Attention (IAA)

**Figure 3:**
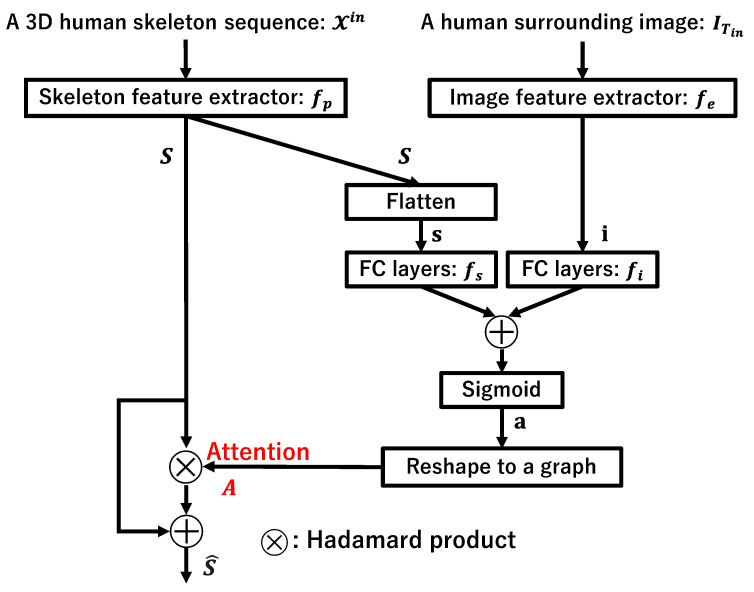
The structure of the IAA module. The skeleton feature extractor is a graph convolution layer of any existing GCN-based prediction model. A surrounding image feature i is used to calculate the attention matrix *A* for modifying the original skeleton feature *S*.

This section explains the details of the proposed skeleton feature modification module, named image-assisted attention (IAA), for future skeleton prediction. The structure of the proposed IAA module is shown in Figure 3.

The IAA module uses a skeleton feature *S* extracted using the first layers of a GCN-based prediction model, namely the skeleton feature extractor $f_{p}$, and a surrounding image feature i extracted using the image feature extractor $f_{e}$ explained in Section 3.2 as input. The feature extraction process is as follows:

(1) $$\begin{array}{cc} S & =f_{p}\left(X^{\mathrm{in}};\Theta_{p}\right). \end{array}$$

(2) $$\begin{array}{cc} i & =f_{e}\left(I_{T_{in}}\right), \end{array}$$

where $\Theta_{p}$ denotes a set of parameters updated during training.

Since the skeleton feature *S* is an output of the first graph convolution layers in a GCN, it is a set of features corresponding to each joint with a graph structure; therefore, first, $S\in R^{m\times n}$ is flattened to a one-dimensional vector $s\in R^{mn}$. Next, s and i are converted by fully connected layers $f_{s}$ and $f_{i}$, respectively, to be the same dimension, *n*-dimensional vectors. Then, these vectors $f_{s}\left(s;\Theta_{s}\right)$ and $f_{i}\left(i;\Theta_{i}\right)$ are added and fed to sigmoid function $\sigma \left(x\right)=\frac{1}{1+\exp(-x)}$ to calculate the attention vector $a\in R^{n}$ as

(3) $$a=\sigma (f_{s}\left(s;\Theta_{s}\right)+f_{i}\left(i;\Theta_{i}\right)).$$

The attention vector a is repeated *m* times to form an attention matrix $A\in R^{m\times n}$. The original skeleton feature *S* is modified by the following equation:

(4) $$\hat{S}=S\circ A+S,$$

where the operator ∘ represents the Hadamard product.

As a result, the skeleton feature *S* with *n*-dimensional features of *m* body joints are weighted by the attention matrix *A*, and the weighted skeleton feature $\hat{S}$ is obtained. These series of calculations make the feature values modified based on the surrounding information extracted from the human surrounding image. This method is different from the well-known cross-attention mechanism, such as using softmax and dot product, but we call it “attention” in this paper because this method enhances the effective features for prediction.

In this study, we used the same vector a as the attention for each body joint, that is, all rows of the attention matrix *A* are the same. This is because we consider calculation costs and the fact that the features of graph convolution involve information about adjacent nodes to each other.

### 3.4. Future Skeleton Prediction Using the Modified Skeleton Feature

Finally, this section explains the future skeleton prediction. The weighted skeleton feature $\hat{S}$ is input to the rest part of the GCN-based model $f_{r}$, and the future skeleton sequence $X^{\mathrm{pred}}$ is predicted as

(5) $$X^{\mathrm{pred}}=f_{r}\left(\hat{S};\Theta_{r}\right),$$

where $\Theta_{r}$ denotes a set of parameters updated during training.

### 3.5. Model Structure and Training

**Figure 4:**
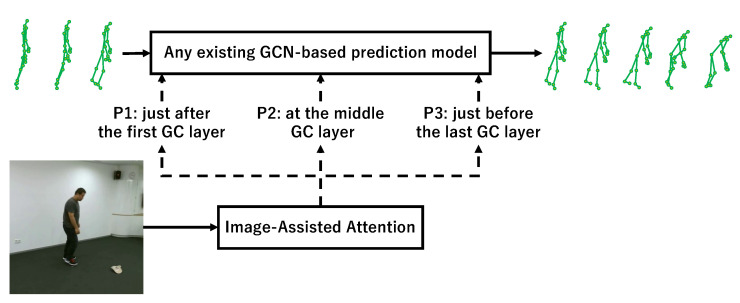
The three locations where the IAA module is inserted into any existing GCN-based model. P1 is the initial stage of the feature extraction, P2 is the end of the feature extraction, and P3 is the final stage of the generating prediction.

In the implementation, there is room to discuss how to divide a GCN-based model into the two $f_{p}$ and $f_{r}$, that is, there are several possibilities to insert IAA into a GCN-based model. In this paper, we considered the three locations for IAA to be inserted: P1: just after the first graph convolution layer of the model, P2: at the middle graph convolution layer of the model, and P3: just before the last graph convolution layer of the model, to investigate and study how the accuracy changes. Here, in the case of encoder–decoder models such as MSR-GCN, we can consider P1 as the initial stage of the feature extraction, P2 as the end of the feature extraction, and P3 as the final stage of the generating prediction. Figure 4 shows the three locations where the IAA module is inserted into any existing GCN-based model.

In Section 4, we will compare the performance by experimentally changing the location of the insertion.

The model training was done in an end-to-end manner. We did not change the loss function from the original GCN-based model we used in the proposed method. The sets of parameters $\Theta_{p}$, $\Theta_{r}$, $\Theta_{s}$, and $\Theta_{i}$ were updated during training.

## 4. Evaluation and Results

### 4.1. Outline of Experiments

To confirm the performance of the proposed method, we performed experiments on publicly available datasets. Here, in the experiments, we used PyTorch 1.12.1 (PyTorch is an open-source software released under the modified BSD license), cuda11 (CUDA is developed by NVIDIA, based in Santa Clara, CA, USA), Python 3.8 (Python is maintained by Python Software Foundation, which is an American nonprofit organization), and NVIDIA Tesla V100 GPUs (Tesla V100 GPUs is developed by NVIDIA, based in Santa Clara, CA, USA).

Since any GCN-based model can be used for the proposed method, we compared several methods. As the GCN-based models, we used the latest prediction method, MSR-GCN [16], and its original method, Traj-GCN [13]. In the evaluation, the proposed method with each GCN-based model is named with the suffix “with IAA”. We used the two GCN-based models with/without IAA; thus, we compared the 2×2 models: MSR-GCN and Traj-GCN, which are existing methods, and MSR-GCN with IAA and Traj-GCN with IAA, which are the proposed methods.

### 4.2. Datasets

To evaluate the future pose prediction models, datasets consisting of 3D human skeleton sequences and corresponding images are required. We used a large-scale dataset, the NTU RGB+D 120 dataset [32], and the PKU-MMD dataset [33].

#### 4.2.1. NTU RGB+D 120 Dataset

**Figure 5:**
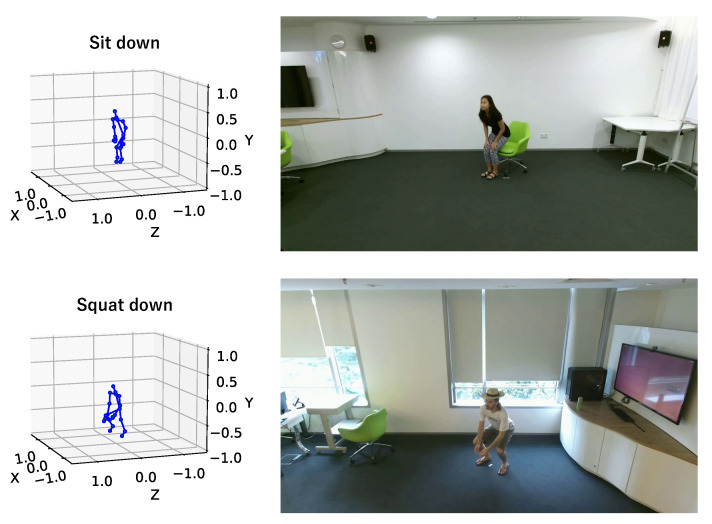
Examples of 3D skeletons and RGB images in the NTU RGB+D 120 dataset. The upper one shows “sit down” and the lower one shows “squat down”. The 3D human skeletons are on the left and the RGB image are on the right.

The NTU RGB+D 120 dataset [32] is basically used to evaluate skeleton-based action recognition since it contains 114,480 videos in 120 action classes. In our experiment, we used 3D and 2D skeleton sequences consisting of 25 joint points for each person and the corresponding 1920×1080 RGB images from the dataset. This dataset is captured using Kinect v2 sensors (Kinect v2 is developed by Microsoft, based in Redmond, WA, USA) at 30 fps. Figure 5 shows some examples of 3D skeletons and RGB images in the NTU RGB+D 120 dataset.We used this for a future pose prediction task. We excluded 535 samples because of incomplete or missing skeleton data.

From this dataset, we generated training and testing data by the following process.

1. Since a sample may contain multiple persons or false detections of other objects, first, we select a person where the variance of the skeleton locations is the maximum.
2. We use 12 frames for 0.4 s as input and 30 frames for 1 s as future prediction by using a sliding window of 42 frames. Multiple subsequences were generated using a sliding window with a stride of 1.
3. The number of joints was reduced to fit the input shape of the prediction models (25 joints → 22 joints). For MSR-GCN, we prepared multi-scale skeletons with 12, 7, and 4 joints by taking the average of the neighboring body joints, according to the preprocessing of the multi-scale joints in MSR-GCN [16].
4. Since the generated subsequences may include switching of persons or other objects, we excluded them by the criterion where the sum of the distance from “head” to “spine base” and from “spine base” to “left foot” is smaller than 60 cm as noise data. Similarly, we also excluded subsequences with skeletons of the distance between any joint and spine base exceeding 140 cm as noise.
5. To make the prediction system robust to the location of the target person, all skeleton sequences are aligned using the locations of “spine base” in the last frame of each input.
6. Images corresponding to the input skeletons were preprocessed by the method described in Section 3.2. Here, the locations of missing body joint values of the 2D skeleton were ignored, and the center points were calculated.
7. We divided the set of subsequences and the cropped images into the training set (50%), validation set (25%), and test set (25%) referring to the cross-subject evaluation method proposed in the NTURGB+D 120 dataset (dividing such that data of the same person is not included in different sets).

#### 4.2.2. PKU-MMD Dataset

**Figure 6:**
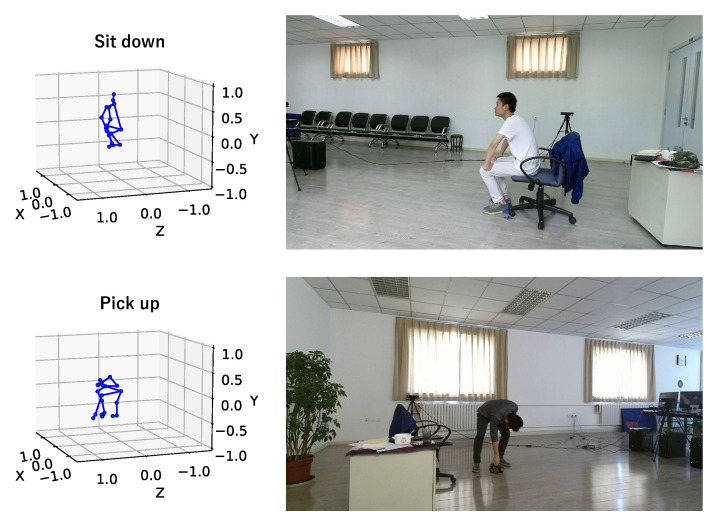
Examples of 3D skeletons and RGB images in the PKU-MMD dataset. The upper one shows “sit down” and the lower one shows “pick up”. The 3D human skeletons are on the left and the RGB image are on the right.

The PKU-MMD dataset [33] is a dataset for continuous multi-modality 3D human action understanding. The PKU-MMD dataset contains two phases: Phase 1, which is a large-margin action detection task, and Phase 2, which is a small-margin action detection task. We used the Phase 2 data in our experiment. Phase 2 contains 2000 short video sequences in 49 action classes, performed by 13 subjects in 3 camera views. In our experiment, we used 3D skeleton sequences consisting of 25 joint points and corresponding 1920×1080 RGB images. Figure 6 shows some examples of 3D skeletons and RGB images in the PKU-MMD dataset.

The procedure of generating training and evaluation data is almost the same as that of NTU RGB+D 120, but the PKU-MMD dataset does not contain 2D skeletons. Hence, we used the object detection model YOLOv5 [34] to detect persons from the RGB images and calculated center points from the bounding boxes. If two or more persons or other objects were detected, the bounding box with the highest confidence level was selected in each frame. Furthermore, if no person was detected in a frame, the bounding box detected in the previous frame was used for the frame. Therefore, these cropped images contain some uncertainty.

### 4.3. Configuration and Parameters

The number of weight parameters for fully connected (FC) layers in the IAA module is shown in Table 1. As shown in Table 1, the number of FC layers in the IAA varied from one to three layers in the experiments.

The other model parameters were set by following the existing studies. Leaky ReLU with a slope of 0.2 was used as the activation function for FC layers $f_{s}$ and $f_{i}$, while the activation function was not applied to the final output of FC layers. All learnable parameters $\Theta_{p}$, $\Theta_{r}$, $\Theta_{s}$, and $\Theta_{i}$ except EffcientNet-B3 were updated by stochastic gradient descent using the Adam optimizer [35], with the learning rate of 0.001 and other hyperparameters set to the PyTorch default settings. The dropout ratio and the maximum number of epochs were set to 0.2 and 200, respectively. In the case of the NTU RGB+D 120 dataset, the batch size was set to 2048. In the case of the PKU-MMD dataset, the batch size was set to 256. When the loss did not decrease for five epochs, the learning rate was multiplied by 0.1. Then, when the validation loss did not decrease for 11 epochs, the training was terminated.

### 4.4. Evaluation Metric

We used mean per-joint position error (MPJPE), which is the mean of all Euclidean distances for each joint in the predicted and ground-truth skeletons, as the loss function and the evaluation metric to evaluate how close the predicted 3D future skeleton sequence is to the ground-truth skeleton sequence. The evaluation metric was proposed in [36] and is widely used in 3D pose prediction and estimation research [13,14,15,16,37,38,39]. The MPJPE (*L*) for a skeleton sequence is defined by the following equation:

(6) $$L=\frac{1}{TJ}\sum_{t=1}^{T}\sum_{j=1}^{J}{∥{\hat{p}}_{j,t}-p_{j,t}∥}_{2},$$

where *J* is the number of body joints in 3D coordinates, *T* is the number of time step, ${\hat{p}}_{j,t}\in R^{3}$ represents the prediction of *j*-th joint location at time step *t*, and $p_{j,t}\in R^{3}$ represents the ground truth of *j*-th joint location at time step *t*.

The training was performed in five trials by changing initial weights for each method, and the performances of each method were evaluated by the average MPJPE of the five trials.

### 4.5. Results

Table 2 shows the average MPJPE of five trials and its standard deviation for each method on the NTU RGB+D 120 dataset. Additionally, Table 3 shows the average MPJPE of five trials and its standard deviation on the PKU-MMD dataset. Here, the MPJPE values are rounded off to the fourth decimal place.

As a result, from Table 2 and Table 3, the proposed methods “with IAA” improved the average MPJPE scores. Here, please recall that P1 is the IAA insertion position just before the first GC layer, P2 is the middle GC layer, and P3 is just before the last GC layer. Hereafter, the models with IAA inserted at P1, P2, and P3 positions are represented by “with IAA P1”, “with IAA P2”, and “with IAA P3”, respectively. From Table 2, we can confirm that both MSR-GCN with IAA P1 and Traj-GCN with IAA P1 with two FC layers have the best average MPJPE among their respective models in the experiment on the NTU RGB+D 120 dataset. Additionally, from Table 3 in the experiment on the PKU-MMD dataset, we can confirm that Traj-GCN with IAA P1 with one FC layer has the highest scores among these Traj-CGN models, while MSR-GCN with IAA P2 with one FC layer has the highest scores among these MSR-CGN models.

**Figure 7:**
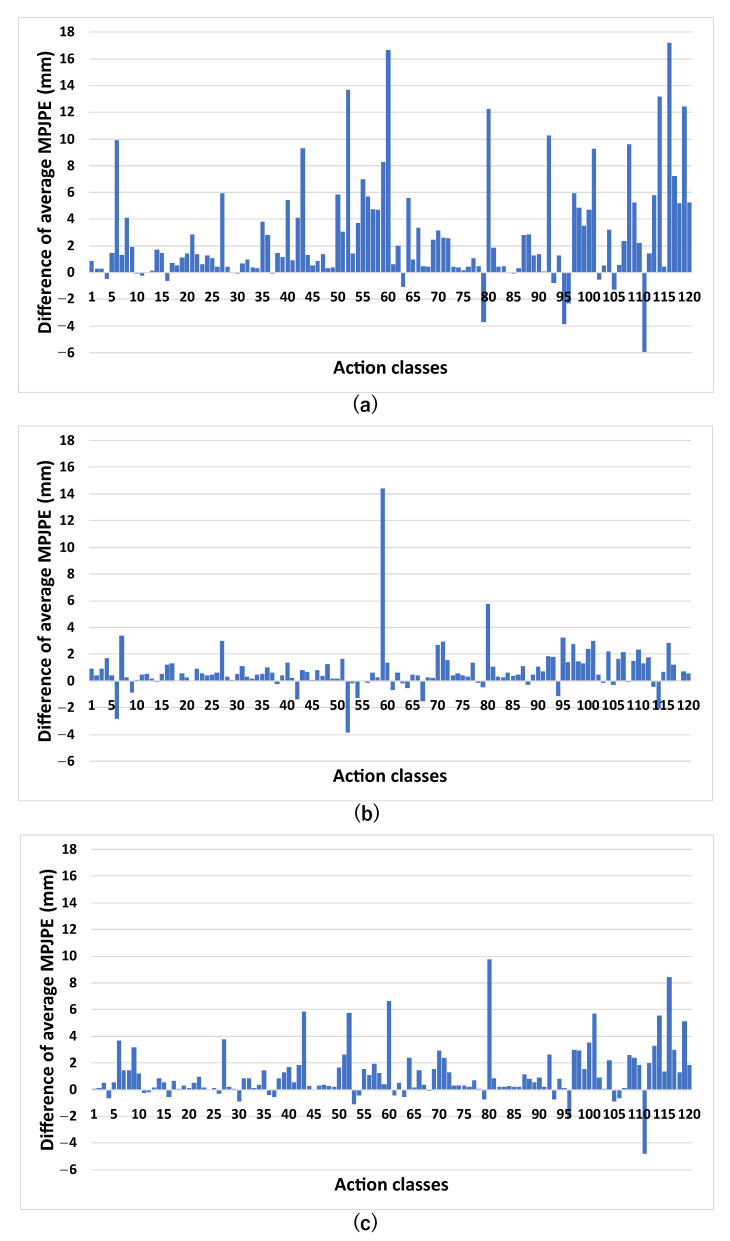
Differences in the class-wise average MPJPE between MSR-GCN and the models of MSR-GCN with IAA calculated. In this figure, (**a**) is the difference of MSR-GCN minus MSR-GCN with IAA P1, (**b**) is the difference of MSR-GCN with IAA P2 minus MSR-GCN with IAA P1, and (**c**) is the difference of MSR-GCN with IAA P3 minus MSR-GCN with IAA P1. Also, MSR-GCN with IAA P1 (2 FC layers), MSR-GCN with IAA P2 (3 FC layers), and MSR-GCN with IAA P3 (2 FC layers) are compared.

Figure 7 shows the difference in the average class-wise MPJPE between MSR-GCN and the models of MSR-GCN with IAA. The classes are 120 action classes labeled in the NTURGB+D 120 dataset. The figure shows the values of the class-wise average MPJPE of each model minus that of MSR-GCN with IAA P1 (two FC layers), which has the highest accuracy. Here, we also compared MSR-GCN with IAA P1 (two FC layers), MSR-GCN with IAA P2 (three FC layers), and MSR-GCN with IAA P3 (two FC layers).

From Figure 7a, we can see that the MSR-GCN with IAA P1 improves the prediction accuracy in many action classes, as the difference of the class-wise average MPJPE is positive in many of the action classes. Looking at each action class, we can see that the prediction accuracy was especially improved for actions involving other persons, such as “walking apart from each other” in action class 60 and “follow other person” in action class 116. In addition, we can also see the improvement of the prediction accuracy for actions “sit down” in action class 8 and “squat down” in action class 80, which can be easily predicted by using the existence of a chair, and actions that are related to other objects such as “pick up” in action class 6 and “move heavy object” in action class 92. In the comparison between the proposed methods, Figure 7b shows that IAA P1 has generally higher accuracy than IAA P2 in each action class, although the difference is small as an overall trend. Furthermore, Figure 7c shows that IAA P1 has generally higher accuracy than IAA P3 in many action classes as the overall trend. The two graphs in Figure 7b,c show that the accuracy of IAA P3 is relatively lower than the other two IAA methods and that the improvement of prediction accuracy is small.

The processing time and frames per second (FPS) required to predict 30 frames from 12 frames of input between MSR-GCN and MSR-GCN with IAA P1 are shown in Table 4. An RTX3090 (RTX3090 GPU is developed by NVIDIA, based in Santa Clara, CA, USA.) was used as the GPU, and CUDA11 and CUDNN libraries (CUDNN is developed by NVIDIA, based in Santa Clara, CA, USA) were used for this speed test. Each processing time includes the time required for preprocessing of the skeleton sequence and the surrounding image. Additionally, the processing time is shown as an average of 1000 times, with three significant digits. In Table 4, the time required for the prediction was less than 0.04 s, although it took four times longer than when only MSR-CGN was used.

**Figure 8:**
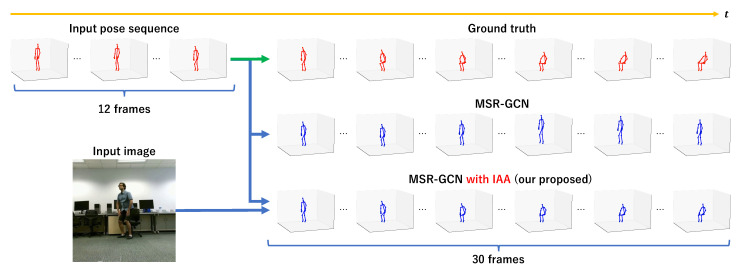
An example of predicted result (blue) and ground truth (red) in the “sit down” action. We can see that the predicted skeletons of MSR-GCN with IAA (with 2 FC layers) are closer to the ground truths when using the image of the surroundings.

**Figure 9:**
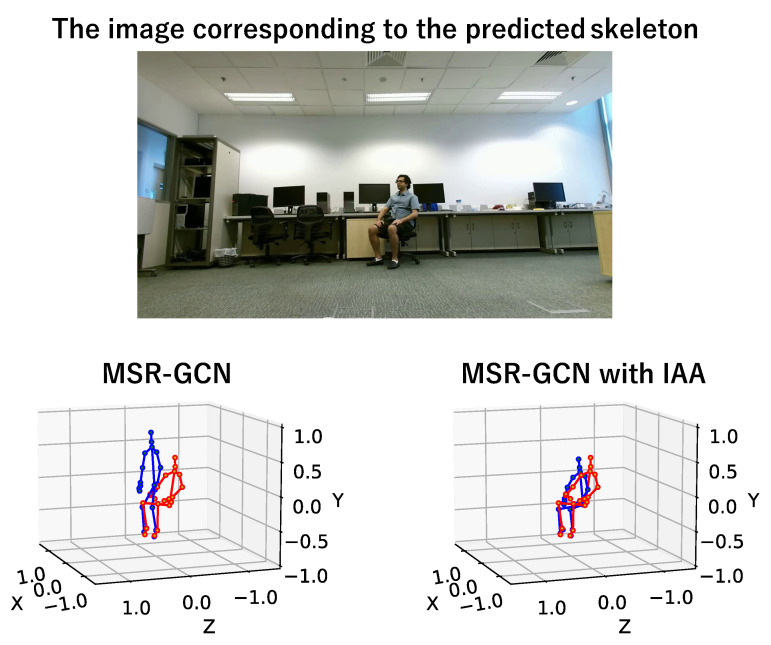
A prediction example of the 30th frame of prediction (blue) and ground truth (red) in “sit down”. In this example, the existing method made the misprediction as “jump up”, but our proposed method predicted it correctly. In object-related motions, the future skeleton is well predicted by our proposed IAA.

**Figure 10:**
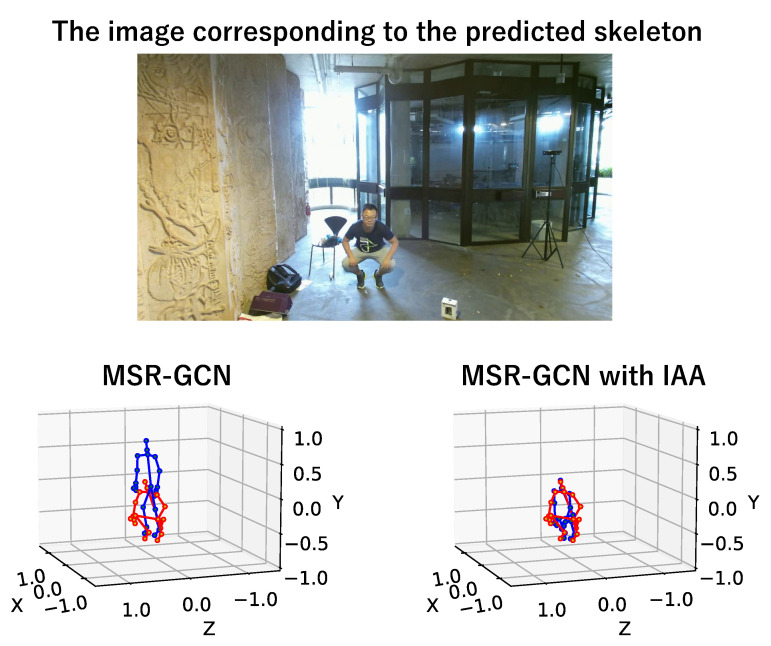
A prediction example of the 30th frame of prediction (blue) and ground truth (red) in “squat down”. In non-object-related motions, the future skeleton is also well predicted by our proposed IAA.

Figure 8 shows an example of the predicted results and the ground truth in the “sit down” class of the NTU RGB+D 120 dataset. Figure 9 also shows the predicted future skeleton at the 30th frame in the output. As another example, Figure 10 shows the result that is the predicted skeleton at the 30th frame in an example of the “squat down” class of the NTU RGB+D 120 dataset.

From Figure 8 and Figure 9, we can also see that our proposed method improves prediction accuracy over existing methods in the “sit down” class, confirming the effectiveness of using the surrounding situation as additional information in object-related motions. It is the same in non-object-related motions, as shown in Figure 10.

### 4.6. Discussion

In Table 2 and Figure 7, IAA P1 achieved the highest accuracy. We assume it is because MSR-GCN and Traj-GCN structures have shortcut connections between each layer; the effect of feature modification worked effectively for all GC layers by weighting in the early stage of feature extraction. However, in Table 3, the prediction was more accurate when IAA is applied to the P2 position for MSR-GCN than when IAA is applied to the P1 position. The MPJPE difference between IAA P1 and IAA P2 is also small in Figure 7, indicating that it is possible for some cases to be more accurate when applied to the P2 position. Therefore, as for the optimal IAA location, since many recent deep learning models have shortcut connections or similar structures, we consider that IAA is basically best applied to the first graph convolution layer of many prediction models; however, in some cases, it may be necessary to consider applying it to the middle of the model.

The IAA improved the prediction accuracy for many motions. However, looking at the results in detail, even though the actions are the same, actors in some videos pretended to perform an action without objects. In such cases, the prediction accuracy was decreased. Additionally, in some actions such as “hands up (both hands)” in action class 95 in Figure 7a, the prediction accuracy was decreased. We guess it is because the actors in the dataset are performing the action while there are many objects on the floor and desks that are not related to the action. In the future, it will be necessary to improve the method in some way, for example, by using distance information between humans and objects to prevent the prediction accuracy from decreasing even in environments where there are many objects unrelated to the actions.

## 5. Conclusions

In this study, we proposed the method to predict future human skeleton sequences from short-term 3D human skeleton sequences with surrounding information as an additional clue, under the assumption that the surrounding information helps the prediction of human motions. The human surrounding information was extracted from an image of the target person. In the experiment using the NTU RGB+D 120 dataset and PKU-MMD dataset, we confirmed that the prediction accuracy of the future skeleton sequence is improved for the motion involving other objects and humans. Additionally, we proposed a novel skeleton feature weighting method, the image-assisted attention (IAA) module, and investigated the effect of the position where the proposed IAA is inserted into the GCN-based methods. As a result, we confirmed that it is basically best to insert the IAA module just after the first graph convolutional layer of a GCN-based prediction model, although it is better when inserted in the middle layer of the model in some cases. In the proposed method, we used EfficientNet for image feature extraction; but there is a possibility that the accuracy will be improved by replacing it with another backbone. We will tackle the remaining issues and further improve the prediction accuracy in the future.

## Figures and Tables

**Table 1:** The number of parameters for fully connected (FC) layers in our proposed IAA module.

**1 layer**	**Input of $f_{s}$** **(1st)**	**Input of $f_{i}$** **(1st)**	**Output of FC layers**
MSR-GCN with IAA	4224 (= 66×64)	1536	64
Traj-GCN with IAA	16,896 (= 66×256)	1536	256
**2 layers**	**Input of $f_{s}$** **(1st / 2nd)**	**Input of $f_{i}$** **(1st / 2nd)**	**Output of FC layers**
MSR-GCN with IAA	4224 / 2112	1536 / 768	64
Traj-GCN with IAA	16,896 / 8848	1536 / 768	256
**3 layers**	**Input of $f_{s}$** **(1st / 2nd / 3rd)**	**Input of $f_{i}$** **(1st / 2nd / 3rd)**	**Output of FC layers**
MSR-GCN with IAA	4224 / 2112 / 1056	1536 / 768 / 384	64
Traj-GCN with IAA	16,896 / 8848 / 4424	1536 / 768 / 384	256

**Table 2:** Avarage MPJPE of 5 trials and its standard deviation on the NTU RGB+D 120 dataset. P1, P2, and P3 are the position where IAA is inserted in the GCN-based model, as described in Section 4.1. ↓ indicates that the smaller the value, the better. The bold indicates the best scores.

Model	Average MPJPE (mm)↓		
MSR-GCN	104.373±0.398		
Traj-GCN	105.464±0.193		
**P1 model**	**1 FC layer**	**2 FC layers**	**3 FC layers**
MSR-GCN with IAA	102.466±0.248	101.817±0.181	102.039±0.130
Traj-GCN with IAA	103.799±0.295	103.213±0.154	103.338±0.146
**P2 model**	**1 FC layer**	**2 FC layers**	**3 FC layers**
MSR-GCN with IAA	103.498±0.107	102.825±0.199	102.544±0.197
Traj-GCN with IAA	105.132±0.246	104.286±0.545	103.905±0.152
**P3 model**	**1 FC layer**	**2 FC layers**	**3 FC layers**
MSR-GCN with IAA	103.105±0.155	102.859±0.207	103.797±0.461
Traj-GCN with IAA	105.172±0.272	104.790±0.265	104.719±0.192

**Table 3:** Avarage MPJPE of 5 trials and its standard deviation on the PKU-MMD dataset. ↓ indicates that the smaller the value, the better. The bold indicates the best scores.

Model	Average MPJPE (mm)↓		
MSR-GCN	137.454±1.696		
Traj-GCN	136.630±0.617		
**P1 model**	**1 FC layer**	**2 FC layers**	**3 FC layers**
MSR-GCN with IAA	135.241±2.604	133.924±1.452	135.686±2.915
Traj-GCN with IAA	135.130±0.507	135.551±0.791	135.843±0.781
**P2 model**	**1 FC layer**	**2 FC layers**	**3 FC layers**
MSR-GCN with IAA	132.972±2.243	137.299±7.038	138.553±4.499
Traj-GCN with IAA	136.330±0.521	136.284±0.956	136.412±0.878
**P3 model**	**1 FC layer**	**2 FC layers**	**3 FC layers**
MSR-GCN with IAA	136.274±5.689	137.465±3.642	137.193±1.766
Traj-GCN with IAA	136.736±0.806	136.735±0.602	136.224±0.445

**Table 4:** Processing time and frames per second (FPS) required to predict 30 frames from 12 frames of input.

Model	Processing Time (s)	Frames per Second (FPS)
MSR-GCN	$7.83\times 10^{-3}$	127
MSR-GCN with IAA P1	$3.07\times 10^{-2}$	32

## Data Availability

The NTU RGB+D 120 dataset can be downloaded from: https://github.com/shahroudy/NTURGB-D (accessed on 21 November 2022). The PKU-MMD dataset can be downloaded from: https://www.icst.pku.edu.cn/struct/Projects/PKUMMD.html (accessed on 21 November 2022).
